# CAR-T: a potential gene carrier targeting solid tumor immune microenvironment

**DOI:** 10.1038/s41392-021-00812-z

**Published:** 2021-11-11

**Authors:** Minjie Wang, Chaocai Zhang, Xiaobing Jiang

**Affiliations:** 1 Department of Neurosurgery, Union Hospital, Tongji Medical College, Huazhong University of Science and Technology, Wuhan, 430022 China; 2 Department of Neurosurgery, Hainan General Hospital/Hainan Affiliated Hospital of Hainan Medical University, Haikou, 570311 China

**Keywords:** Cancer microenvironment, Tumour immunology

A recent study published in Cell by Lexus et al. presented CAR-T cell as a carrier that could secrete extracellular vesicles (EVs) containing immunostimulatory RNA RN7SL1, which could be specifically taken in by immune cells to enhance endogenous immunity against solid tumor.^1^

Chimeric antigen receptor T (CAR-T) immunotherapy is a novel anti-tumor therapy through transferring genetically engineered T cells expressing a chimeric antigen receptor (CAR) specific for tumor antigens. It has shown remarkable efficacy in treating hematologic malignancies such as leukemia, lymphoma, and myeloma. However, CAR-T cells cannot exert persistent therapeutic effect on some solid tumors due to the cancer heterogeneity and suppressive tumor microenvironment (TME), which cause relapse from cancer cells with CAR antigen loss and rapid CAR-T cells exhaustion, especially for glioblastoma (GBM).^2^ Recent studies constantly aim at improving CAR-T cell therapy by engineering the CAR protein, T cells and the interaction between T cells and other components in the TME. Among them, remodeling TME to promote the endogenous immune response is one of the most attractive way to achieve a lasting CAR-T cells response.^3^ Nowadays, accumulating gene therapies emerge to remodel TME because of the relatively stable gene expression of TME.^4^ However, due to the lack of delivery specificity in most existing carriers (nanoparticles and biomaterials), gene therapy stimulating the immune cells may also promote cancer progression. For example, PRR and IFN signaling in immune cells are immunostimulatory, while the same signaling in cancer cells drives cancer progression and immunotherapy resistance.^1^ Hence, a delivery system specific for immune cells is urgently needed for gene therapy targeting TME.

In a recent study in *Cell*, Lexus et al. put forward that delivering the pattern recognition receptor agonists was an effective way to stimulate anti-tumor immunity.^1^ However, it’s hard to deliver them to immune cells directly and it often initiates cancer cell-intrinsic detrimental pathways on the contrary. Hence, Lexus et al. engineered CAR-T cells to deliver immunostimulatory non-coding RNA RN7SL1 with EVs, which functions as a damage-associated molecular pattern (DAMP) to activate RNA PRRs in immune cells.^1^ Lexus et al. considered that CAR-T cells could kill target cancer cells within the scope of the EVs and thus realize selective tumor-infiltrated immune cells delivery, which avoids the initiation of cancer cell-intrinsic detrimental pathways. In immune cells, RN7SL1 limits the accumulation of myeloid-derived suppressor cells (MDSCs) and inhibits TGF-β1 signaling pathway. Meanwhile, it also promotes the development of inflammatory dendritic cells (DCs) and contributes to an increase in effector-like T (Teff) cells and effector-memory-like T (Tem) cells. Due to the comprehensive endogenous immune activation, CAR-T cells efficacy can be improved and generate a positive immune cycle. Further, Lexus et al. designed to deliver peptide antigens to cancer cells by CAR-T cells and make CAR-T cell therapy persist regardless of CAR antigen loss. This strategy simultaneously employs CAR-T cells, enhances endogenous T cell function, and counteracts common suppressive mechanisms, offering effective combinatorial approaches to improve solid tumor response.^1^

**Fig. 1:**
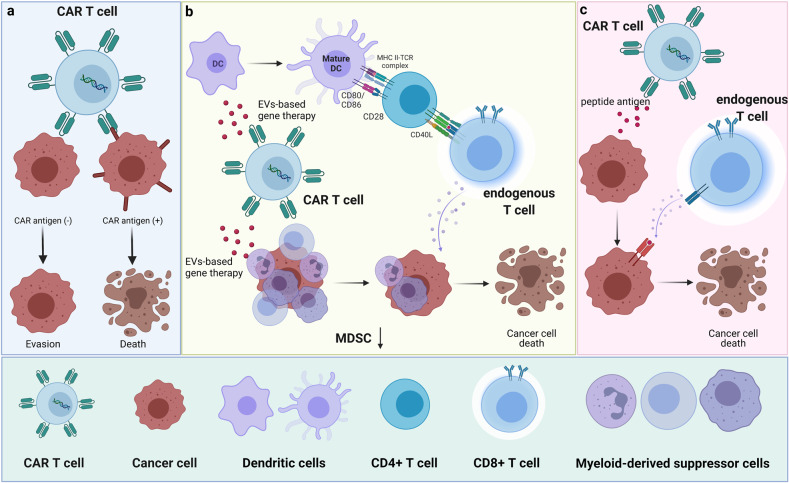
Effects of CAR-T cells delivery system in cancer. **a** CAR-T cells enter in tumor area and cause the first killing: CAR-T cells recognize CAR antigens in a major histocompatibility complex (MHC)-independent manner and mediate secretory killing and non-secretory killing. **b** Reprogramming tumor immunosuppressive microenvironment and stimulating endogenous T cells: CAR-T cells deliver EV-based gene therapy to limit the accumulation of myeloid-derived suppressor cells (MDSCs) and inhibit TGF-β1 signaling pathway. Meanwhile, it also promotes the development of inflammatory dendritic cells (DCs) and an activation of endogenous T cells. **c** Turning “cold tumor” to “hot tumor” for glioblastoma: CAR-T cells deliver peptide antigen to cancer cells with CAR antigen loss and totally relieve the immune escape of tumor cells through endogenous immunity

T cells have good deformability and can penetrate the blood-brain barrier under certain conditions, and T cells with specific CAR antigens (IL13Rα2, EGFRvIII, and HER2) can kill target GBM cells to a large extent despite the effect is not lasting. These all equip CAR-T cells delivery system with the potential to shift the treatment landscape and outlook for patients with GBM. First, CAR-T cells enter in tumor area and cause the first killing (Fig. 1a). Meanwhile, CAR-T cells secrete pro-inflammatory signaling molecules to immune cells and achieve effective adaptive immunity at the proper time. Gradually, the TME is reprogrammed and endogenous T cells of multiple specificities can be stimulated to yield a polyclonal anti-tumor response, which initiates secondary killing and forms a positive anti-tumor cycle from CAR-T cells to T cells (Fig. 1b). Moreover, CAR-T cells deliver peptide antigen to cancer cells with CAR antigen loss and make them recognized and killed by endogenous T cells, which totally relieves the immune escape of tumor cells and may turn “cold tumor” to “hot tumor” for GBM (Fig. 1c). Nowadays, amounts of researches on protein molecules, mRNA, and noncoding RNA have been performed to figure out their role in the GBM immune microenvironment, but the ways for their clinical application are limited.^5^ This research from Lexus et al. just provides a new perspective for clinical transformation, taking immunotherapy for GBM to the next level. In the future, more substances can be loaded by CAR-T cells for different purposes. For example, CAR-T cells may delivery SN-38, an active metabolite of irinotecan killing tumor cells but not immune cells, to achieve widely GBM killing effect; and CAR-T cells may carry PD-1 monoclonal antibody to specifically work on immune cells, increasing the sensitivity to PD-1 monoclonal antibody.

In summary, Lexus et al. have demonstrated that the gene delivery by CAR-T cells possesses multiple advantages over other therapeutic delivery methods in terms of timing and space distribution. As CAR-T cells did not show optional effects on some solid tumors especially for GBM, we thought the core purpose of CAR-T cell therapy should be transferred from single cancer killing to being a specific gene carrier targeting the TME. We believe that these findings will expand our understanding of CAR-T cells engineering, and point to future combinations of gene therapy and CAR-T cell therapy in GBM research and clinical management.
